# Biostimulant Properties of Seaweed Extracts in Plants: Implications towards Sustainable Crop Production

**DOI:** 10.3390/plants10030531

**Published:** 2021-03-12

**Authors:** Omar Ali, Adesh Ramsubhag, Jayaraj Jayaraman

**Affiliations:** Department of Life Sciences, Faculty of Science and Technology, The University of the West Indies, St. Augustine, Trinidad and Tobago; omarali0418@gmail.com (O.A.); Adesh.Ramsubhag@gmail.com (A.R.)

**Keywords:** seaweed extracts, phytoelicitor components, phytostimulation, stress tolerance, mechanisms of action, organic inputs, sustainable agriculture

## Abstract

The use of seaweed-based bioproducts has been gaining momentum in crop production systems owing to their unique bioactive components and effects. They have phytostimulatory properties that result in increased plant growth and yield parameters in several important crop plants. They have phytoelicitor activity as their components evoke defense responses in plants that contribute to resistance to several pests, diseases, and abiotic stresses including drought, salinity, and cold. This is often linked to the upregulation of important defense-related genes and pathways in the plant system, priming the plant defenses against future attacks. They also evoke phytohormonal responses due to their specific components and interaction with plant growth regulation. Treatment by seaweed extracts and products also causes significant changes in the microbiome components of soil and plant in support of sustainable plant growth. Seaweed extracts contain a plethora of substances which are mostly organic, but trace levels of inorganic nutrient elements are also present. Fractionation of seaweed extracts into their components and their respective bioassays, however, has not yielded favorable growth effects. Only the whole seaweed extracts have been consistently proven to be very effective, which highlights the role of multiple components and their complex interactive effects on plant growth processes. Since seaweed extracts are highly organic, they are ideally suited for organic farming and environmentally sensitive crop production. They are also very compatible with other crop inputs, paving the way for an integrated management approach geared towards sustainability. The current review discusses the growth and functional effects evoked by seaweed extracts and their modes and mechanisms of action in crop plants which are responsible for elicitor and phytostimulatory activities. The review further analyses the potential value of seaweed extracts in integrated crop management systems towards sustainable crop production.

## 1. Introduction

Research efforts have been undertaken over decades to find various workable organic agricultural inputs that are not only beneficial to humans but also remain sustainable towards the environment. With the onset of climate change, pesticide resistance, and the continuous loss of land due to overgrowing populations, the need for new innovative agricultural practices is paramount more than ever. 

The term biostimulants refers to substances of biological origin or microorganisms which, when applied to plants either via root drench, foliar spray, or a combination of both, is intended to stimulate natural processes in the plant that is responsible for efficient plant nutrient use efficiency and growth processes and/or an increase in the tolerance to abiotic and biotic stress, irrespective of the plant-beneficial nutrient content of the substances [1,2]. Algal extracts are biostimulants rather than fertilizers since they stimulate defense and growth response when applied to the plant. Furthermore, the profiles of algal extracts have not been shown to naturally contain fertilizer compounds at the level to qualify it as a fertilizer. Much focus has been placed on seaweed-based extracts recently since studies revealed that these complex mixtures possessed assorted biostimulatory compounds such as various forms of carbohydrates, amino acids, small quantities of phytohormones, osmoprotectants, and proteins [3,4]. In addition to enhancing stress tolerance, nutrient uptake, growth, and yield, seaweed-based biostimulants have also been shown to help reduce seed dormancy and enhance root systems, flowering [5], fruit quality, and taste [6], and even the quality of produce [7]. These all-round effects lead to improved crop productivity. 

Seaweeds are macroalgae which constitute an integral component of marine and coastal ecosystem, contributing to their rich biodiversity and to the overall biosphere. There are three classes of seaweeds based on their color, which have been commercially utilized for various purposes including agriculture (Table 1). Some of the seaweeds are abundantly available and also commonly found irrespective of the geographical location, though some of them are specific to certain regions. During recent years, there has been a huge influx of Sargassum in many parts of the Americas and the Caribbean. Although massive quantities of the seaweed being deposited on the coasts frequently create environmental crises, there is also an opportunity for undertaking innovative actions for the valorisation of this biomass, rather than simply discarding in landfills or leaving it to decay. This could be very well realized by the production of seaweed-based biostimulants and other bioproducts. Interestingly, seaweed extracts have repeatedly been shown to contribute to plant growth promotion, increased yields, and plant’s tolerance to abiotic and biotic stresses [8]. This is certainly a promising and sustainable approach that farmers can incorporate in to their farming systems, even in integrated crop management whereby efforts can be made to minimize chemical pesticide usage by replacing the synthetic inputs with seaweed extracts/products. Furthermore, efforts have been made to understand comprehensively how these seaweed extracts work at influencing such an increase in overall crop productivity via the global exploration of differential gene expression. The question of where these seaweed-based biostimulants stand is still pertinent to some. In this respect, the current review dives into the effects of seaweed extracts on economically important crops which will then be synchronized with the mechanism of action at the genetic and metabolic planes. The review will portray the usage of these extracts in complementation with marginal levels of chemical inputs to produce the most sustainable outcome in agriculture, both economically and environmentally. 

## 2. Seaweed Extracts—Methods of Preparation and Application into Plants

Both physical methods (heat, pressure, and microwaves) and chemical methods (solvents, acids, and alkali) are used for the extraction of seaweeds. The choice of extraction method should be able to deal with the complexity of the seaweed composition and guarantee the integrity of biologically active molecules that have biostimulant value. The most widely used extraction process involves alkaline extraction at high pressure. This method has been found to be optimally effective, although some hormonal molecules can be degraded. The advantage of this method is the high level of extractability and moderate degradation of polysaccharides into oligomers which are one of the most biologically active components of seaweed extracts [9,10].

The method of application of the seaweed extracts plays an important role in their use and responses by plants. Most application types are either foliar, root application, or a combination of both. The extracts can be applied to soil or growing medium through fertigation, drenching or dripping [11]. However, foliar sprays of less than or equal to 0.05% v/v of the extract have been reported to be optimal for the crop and result in more effective control of disease and higher yields [12]. The better performance of foliar applications has been attributed to the immediate interaction with the plant tissues because foliar absorption happens almost immediately. Further, the adsorption of extracts by soil particles is common, which may reduce its instant mobility [12,13]. Additionally, the optimal application times for these extracts were determined to be around every 10–14 days for provoking the best plant responses [14].

## 3. Effect of Seaweed Extracts on Plant Growth

Over the decades, seaweed extracts have been highly explored for possible use in crop production for improving biomass yield and produce quality. These extracts have been shown to positively affect seed germination and plant growth at all stages up to harvest and even post-harvest [5,13] (Table 2). Seaweed products have been shown to promote increased germination rates and cause significant increases in seedling vigor by enhancing root size and density [15]. The extracts have also been shown to protect seedlings from transplantation shock in tomato, cabbage, and marigold [16,17]. The improved rooting architecture could be a result of small levels of phytohormones present in the extracts such as auxins as well as various stimulatory processes engaged in the plant system upon treatment with these extracts [18]. The enhancement of root systems of plants treated with seaweed extracts was also observed in vegetatively propagated plants. For instance, cuttings from floricultural plants such as marigold treated with an extract from *E. maxima* led to an increase in root density [19]. This was also reported in stone pine cuttings treated with *E. maxima* extract which is otherwise very difficult to root [20]. Treatment with *A. nodosum* extract was able to increase the number of propagules per plant in daylilies [21]. Extracts of *A. nodosum* and *K. alvarezii* also improved water uptake and nutrients, which ultimately led to the promotion of overall vigor and the growth of plants [22,23]. Application of *A. nodosum* and *Laminaria* spp. extracts in maize showed that leaves were able to significantly absorb more Zn, Fe, B, Cu, Mo, S, Mg, Ca, and Mn than the controls [24]. Applications of *A. nodosum* on cottonwood significantly increased potassium uptake in the leaves [25]. A similar increase in potassium levels was also observed in the leaves of mustard treated with *E. maxima* [26].

Seaweed extracts are also reported to have or influence phytohormonal activity. Results of a study in spinach treated with *E. maxima* extracts showed the increase of plants’ endogenous cytokinins, isopentyladenine, dihydrozeatin, and cis-zeatin which have all been linked to positive plant growth [27]. At the plant’s vegetative stage, application of *A. nodosum* in tomato and sweet pepper led to the increased chlorophyll content of leaves which was probably due to inhibition of chlorophyll degradation caused partly by betaines present in the extract [28,29]. These betaine compounds in the seaweed extracts suspend photosynthetic activity loss by the inhibition of chlorophyll degradation [30]. Similarly, a significant increase in chlorophyll content, stomatal conductance, photosynthetic rate, and transpiration rates were recorded in asparagus plants treated with *A. nodosum* [31]. Treatment of willow plants with an extract of *E. maxima* enhanced the electron transfer rates of both photosystems [32]. Tomato plants treated with red, brown, and green species of seaweed extracts resulted in increased plant height, increased leaf numbers, increased root width and root length, and an overall increase in biomass [5,33]. 

Seaweed extracts also triggered early flowering and increased fruit set in a variety of crop plants, for example, tomato, pepper, and snap bean [5,12,34,35]. These increases in flower numbers and fruit set inevitably led to an improvement in yields. For instance, the application of seaweed extracts in tomatoes caused a significant increase in flower number, inflorescence number, flower to fruit ratio, and increased fruit number and size [26,28]. This yield increase was thought to be as a result of various levels of phytohormones present in the extracts such as cytokinins and induction of host hormonal synthesis [36]. Recent studies have shown that seaweed extracts and their components can modulate the expression of genes responsible for the endogenous biosynthesis of growth hormones including auxin, cytokinin, and gibberellin [5]. This was reported in tomato and sweet pepper plants treated with extracts of *A. nodosum*, *S. vulgare*, and *A. spicifera* [5,13]. Apart from increasing harvestable crop yield, extracts have been reported to enhance the nutrient quality of crops such as tomato, pepper, lettuce, spinach, cucumber, and strawberry [6,12,37,38,39]. Treatment of cucumber with an extract of *Macrocystis pyrifera* led to significant increases in total phenols, antioxidant capacity, and vitamin C in the fruits [39]. Applications of *A. nodosum* lead to increases in anthocyanins and total phenolic contents in the grapevines and berries [40]. Strawberry plants treated with *A. nodosum* seaweed extract improved the edible quality of the fruit by enhancing total soluble solids, sucrose, and fructose. The same study also reported an increase in a health compound, quercetin, which has been highly documented as a cardiovascular promoter and anticancer-reducing agent [6]. Application of an extract of *Codium tomentosum* as a postharvest spray to apples resulted in a reduced browning index coupled with inhibition of peroxidase and polyphenol oxidase, enzymes linked to browning which can reduce the shelf life of the produce [41]. These booster effects have been shown to accumulate in the plant irrespective of the type of application done, i.e., foliar, soil root drench, or a combination of both [12,42,43]. Ashing of the seaweed extract product leads to the loss of biostimulant activity which confirmed the role of organic fraction of these seaweed extracts in eliciting positive growth responses in plants [42]. Though seaweed extract biostimulants contain minimum levels of minerals that plants can readily assimilate, the main contribution of the extracts is their ability to stimulate various processes in the plant system which would eventually allow for enhanced growth and productivity of plants [5,44,45]. 

## 4. Effect of Seaweed Extracts on Plants’ Tolerance to Biotic Stresses

The ever-changing climate and the extensive overuse of chemical pesticides have increased the emergence of infectious and resistant pests and pathogens in major crops, thus substantially reducing agricultural outputs [46,47]. Nematode parasites cause serious infestation and damage to plants; however, seaweed extracts have been shown to reduce the infestation of nematodes in plants such as *Arabidopsis thaliana* [43], sunflower [44], and tomato [45]. However, this nematocidal activity is largely a part of the plant’s defense response, possibly by cytokinin: auxin ratio adjustments as it was shown that seaweed extracts had no direct nematicidal properties [48]. Furthermore, extracts of *Sargassum wightii* and *Padina pavonica* showed significant insecticidal activity against the red cotton stainer (*Dysdercus cingulatus*) which is a serious pest harming cotton crops [49]. The infestation of greenfly aphid (*Aphis gossypii*) and serpentine leafminer (*Liriomyza trifolii*) was also significantly reduced in cotton upon mixed treatments with S*argassum* spp., *A. nodosum*, *Laminaria* spp. [50]. On the other hand, citrus greening was also reduced by a reduction of the pest *Diaphorina citri* upon treatment with extracts of *Caulerpa sertularioides*, *Laurencia johnstonii* and *Sargassum horridum* [51]. Additionally, seaweed extracts were able to significantly reduce infestation caused by borers, aphids, and thrips in sugarcane thus preventing great economic loss [52,53]. This reduction in infestation can be due to the antifeedant effects, growth inhibition, and also cytotoxicity on ovarian tissue cells of the pests. For example, an acyclic diterpenoid isolated from *Sargassum* had growth repellent effects against pink bollworm [54]. 

Seaweed extracts also serve as elicitors to plant defense responses against harmful bacterial, fungal, and even viral pathogens thereby protecting crops from major economic damage from diseases [4,25]. Extracts of various brown, red, and green macroalgae (Table 2) were recorded to have great eliciting effects against some harmful bacterial and fungal pathogens. There were several fungal and bacterial diseases that were controlled by the application of seaweed extracts. The reduction of infection levels is due to a general improvement of vigor of seaweed extract treated plants, preformed resistance, induced systemic or systemic acquired resistance, or enhanced soil suppressiveness due to altered microbial dynamics.

Apart from eliciting defense towards bacterial and fungal pathogens, seaweed extracts have shown the potential to control the harsh symptoms of viroid and viruses of plants [55]. Symptoms of the tomato chlorotic dwarf viroid were significantly decreased when pre-treated with a λ-carrageenan polysaccharide from a seaweed extract. Similarly, the severity of tobacco mosaic virus (TMV) in tobacco was significantly reduced when treated with sulfated galactans which are a major component of some of the seaweed extracts [55]. Furthermore, treatment of plants with oligosaccharides derived from seaweeds showed a significant reduction of symptoms caused by tobacco mosaic virus in tobacco plants [56,57]. 

## 5. Effect of Seaweed Extracts on Plants’ Tolerance to Abiotic and Environmental Stresses

In addition to stresses caused by pests and diseases, various environmental stresses such as drought, high temperature, salt, and freezing conditions can hamper crop productivity. It is also estimated that by the year 2050, approximately 50% of arable lands will be plagued by high salt and drought conditions. These abiotic stresses can lead to the build-up of reactive oxygen species (ROS) which will ultimately cause damage to the plant system [56,57]. Interestingly, plants treated with seaweed extracts such as *A. nodosum* and *Sargassum* spp. were able to withstand the damaging effects of these abiotic stresses (Table 2). For example, a significant reduction was recorded in leaf osmotic potential when grapevines and tomato plants were treated with seaweed extracts thus preventing extensive damage [58]. *Kappaphycus alvarezzi* extract treatment on various wheat varieties under salinity and drought stress resulted in plants with increased root length, enhanced chlorophyll content and carotenoids, and tissue water content. The extract also caused a significant reduction in electrolyte leakage and lipid peroxidation, decreased Na^+^/K^+^ ratio, and increased Ca content, thereby reducing ionic disparity. Further, treated wheat plants accumulated osmoprotectants including proline, amino acids, and total protein [59]. Seaweed extracts also promote freezing tolerance in barley [60] and *A. thaliana* [61] with an increase in winter hardiness when treated with seaweed extract sprays. Seaweed extract-induced attenuation of the harsh effects of drought, cold, and salinity stress has shown to be mediated through enhanced root morphology, a build-up of non-structural carbohydrates which improved storage of energy, enhanced metabolism, and water adjustments, as well as the build-up of proline [60,61]. 

The enhancement and priming effects of seaweed extracts on the plant’s defenses against both abiotic and biotic stresses can be attributed to the chemical composition of the extracts as well as its eliciting properties [1]. In the subsequent sub-sections, we will reveal the proposed modes of action of seaweed extracts in eliciting growth and defense responses as well as discuss their compositional features in relation to the varying responses recorded [9]. 

**Table 2:** Effect of seaweed extract biostimulants on major crops.

Crop	Seaweed Extract	Observed Effects	Reference
Tomato (*Solanum lycopersicum*)	- *Ascophyllum nodosum* - *Sargassum* spp. - *Cystoseira myriophylloides* - *Gelidium serrulatum* - *Ulva lactuca* - *Laminaria digitata* - *Fucus spiralis* - *A. spicifera*	- Increased germination rate and seedling vigor - Increased shoot and root growth - Increased chlorophyll content (Soil Plant Analysis Development—SPAD index) - Increased flowering - Fruit yield increase - Fruit quality improvement - Improved resistance to pathogens: Verticillium wilt (*Verticillium dahliae*), early blight (*Alternaria solani*), crown gall (*Agrobacterium tumefaciens*), and bacterial spot (*Xanthomonas campestris* pv. *vesicatoria*) - Increased tolerance to salinity, drought, and cold stress	[3,11,31,32,62,63,64,65,66,67]
Sweet pepper (*Capsicum annuum*)	- *A. nodosum* - *Sargassum* spp. - *A. spicifera*	- Increased shoot and root growth - Increased chlorophyll content (SPAD index) - Increased flowering - Fruit yield increase - Fruit quality improvement - Improved resistance to pathogens: early blight (*Alternaria solani*), blight and fruit rot (*Phytophthora capsica*) and bacterial spot (*Xanthomonas campestris* pv. *vesicatoria*) - Increased tolerance to salinity and drought stress	[3,11,60,61,63,68]
Lettuce (*Lactuca sativa*)	- *A. nodosum* - *Durvillaea potatorum* - *Durvillaea antarctica* - *Ecklonia maxima*	- Increased root and shoot - Increased chlorophyll content - Increased photochemical efficiency and increased activity of photosystem II - Marketable yield increase	[37,68]
Cauliflower (*Brassica oleracea*)	- *A. nodosum*	- Increased heart size - Increased curd diameter	[69]
Soybean (*Glycine max*)	- *A. nodosum* - *Kappaphycus alvarezii*	- Improved nutrient uptake - Enhanced yield parameters - Improved drought tolerance	[70,71,72]
Strawberry (*Fragaria x ananassa*)	- *A. nodosum* - *Sargassum* sp. - *Laminaria* sp. - *Duvillaea potatorum*	- Increased vegetative growth - Increased crown carbohydrate, leaf phosphorus, and potassium contents - Increased yield - Enhanced fruit quality and taste - Increased resistance to powdery mildew (*Podosphaera aphanis*), grey mold (*Botrytis cinerea*), leak (*Rhizopus* and *Mucor* spp.), anthracnose (*Colletotrichum acutatum*), leather rot (*Phytophthora cactorum*), and stem end rot (*Gnomonia comari*)	[6,72,73,74,75]
Cucumber (*Cucumis sativus*)	- *A. nodosum* - *Macrocystis pyrifera* - *Ulva armoricana*	- Increased fruit yield - Enhanced nutritional fruit content - Reduced fungal infections by leafspot (*Alternaria cucumerinum*), blight (*Didymella applanata*), wilt (*Fusarium oxysporum*), grey mold (*Botrytis cinerea*), and powdery mildew (*Erysiphe polygoni*, *E. necator* and S*phareotheca fuliginea*)	[76,77,78]
Onion (*Allium cepa*)	- *A. nodosum*	- Increased germination rate and seedling vigor - Increased bulb diameter and weight - Increased mineral content - Increased ascorbic acid - Disease reduction caused by downy mildew (*Peronospora destructor*) - Aided in water stress resistance and increased N, P, K uptake	[79,80,81,82]
Potato (*Solanum tuberosum*)	- *A. nodosum* - *K. alvarezii* - *Gracilaria edulis* - *E. maxima*	- Growth improvement - Increased yield and tuber quality - Increased resistance to drought stress	[83,84,85,86,87]
Broccoli (*Brassica oleracea* var. *italica*)	- *A. nodosum*	- Increased biomass - Increased nutritional value	[87,88]
Spinach (*S. oleracea*)	- *A. nodosum* - *E. maxima* - *Codium liyengarii*	- Increased fresh yield, dry biomass and leaf area - Increased SPAD index - Increased micro/macronutrient profile - Increased resistance to drought stress	[34,89,90,91]
Carrot (*Daucus carota*)	- *A. nodosum* - *E. maxima*	- Increased harvest index (HI) - Improved nutritional content - Reduction of fungal disease severity caused by black rot (*Alternaria radicina*) and grey mold (*B. cinerea*)	[92,93,94,95,96]
Wheat *(Triticum aestivum*)	- *A. nodosum* - *E. maxima* - *K. alvarezii* - *G. edulis* - *G. dura* - *Sargassum latifolium* - *Ulva lactuca*	- Increased chlorophyll content (>SPAD) - Increased yield - increased micro/macronutrients in root, leaves, and grains - Increased protein content - Improved drought and salinity tolerance	[43,97,98,99,100,101,102]
Rice (*Oryza sativa*)	- *A. nodosum* - *Kappaphycus* sp. - *Gracilaria* sp. - *Hydroclathrus* sp. - *Sargassum* sp.	- Greater germination % and seedling vigor - Improved yield - Improved nutrient uptake	[103,104,105,106]
Apple (*Malus domestica*)	- *A. nodosum* - *Codium tomentosum*	- Decreased alternate bearing - Greater chlorophyll content - Increased photosynthesis and respiration rates - Increased fruit set and fruit yield - Increased anthocyanin content - Improved red color intensity - Minimized fruit browning post-harvest	[39,107,108,109,110]
Maize (*Zea mays*)	- *A. nodosum* - *Laminaria* sp. - *Gracilaria edulis* - *K. alvarezii*	- Increased germination % and rate - Increased seedling vigor - Increased shoot and root growth - Increased net carbon assimilation - Total grain yield	[22,111,112,113,114,115]
Orange (*Citrus* spp.)	- *A. nodosum* - *E. maxima* - *Sargassum horridum* - *Laurencia johnstonii* - *Caulerpa sertularioides*	- Increase in maturity index (MI) - Lessened fruit drop - Increased yield - More vitamin C - Increased TSS - Significant control of the Asian citrus psyllid, *Diaphorina citri*	[14,49,116,117,118]
Sugarcane (*Saccharum officinarum*)	- *A. nodosum*	- Increased biomass in plantlets - Increased plant growth parameters (SPAD, height and leaf area index) - Increased yield - Improved sugar content - Enhanced water retention capacity and water content - Lowered risk of wilting - Significant control of borers, aphids, and thrips	[53,118,119]

## 6. Mechanisms and Mode of Biostimulatory Activities

**Figure 1:**
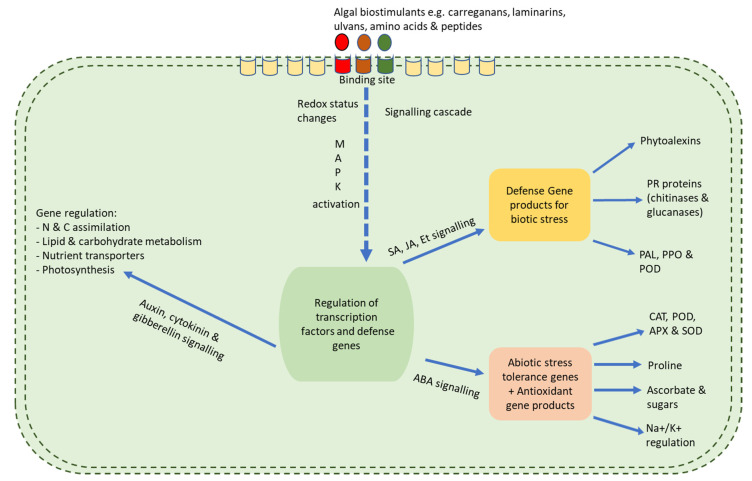
A schematic diagram outlining the mechanism of action of seaweed extract-based biostimulants. When the elicitor binds to the algal receptor sites, it leads to the activation of secondary messengers which causes a myriad of downstream processes [94]. After perception of an elicitor, reversible phosphorylation and dephosphorylation of the plasma membrane proteins and cytosolic proteins occur; this follows cytosolic (Ca^2+^) enhancement; Cl^−^ and K^+^ efflux/H^+^ influx; alkalinization of the extracellular membrane and acidification of the cytoplasm with the activation of the mitogen-activated protein kinase (MAPK). Following MAPK activation, the production of reactive oxygen species (ROS) and reactive nitrogen species (RNS) occurs as well as NADPH oxidase initiation [137,141,142]. When this occurs, a cascade of chemical reactions in the plant which then allows for resistance and enhanced growth regulation.

**Figure 2:**
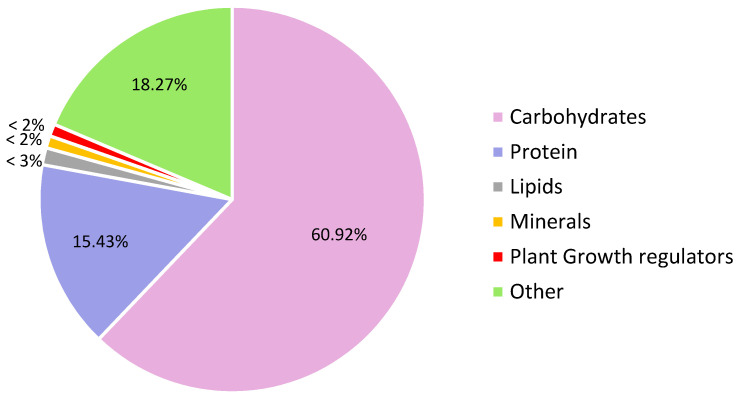
The estimated composition of seaweed extracts belonging to the three mega classes of seaweeds (red, green and brown).

**Figure 3:**
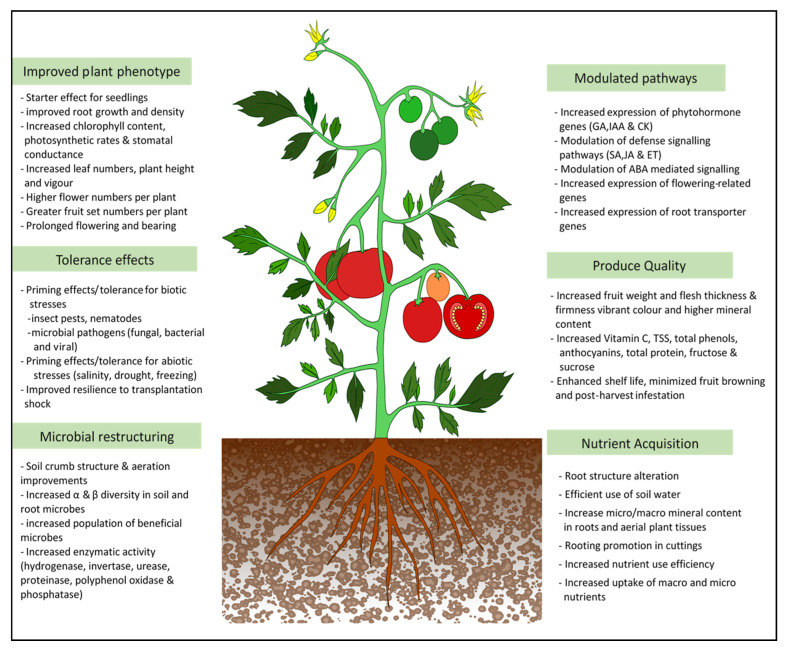
Overview of the positive effects of seaweed extracts on the plant and soil systems.

Seaweed extracts have many growth benefits when applied to plants. However, these benefits are mostly due to their stimulatory feature which causes a cascade of reactions within the plant, thereby leading to overall growth and improvement to resist both biotic and abiotic stress (Figure 1). This section looks at the mode of biostimulatory action of seaweed extracts. However, it should be carefully noted that because seaweed extracts contain a myriad of bioactive ingredients, no one particular component can be allotted to the positive benefits observed (Figure 2; Table 3). In fact, trials using fractions of seaweed extracts have reported that no single fraction was able to replicate all the effects seen when the whole original extract is used. This trend, therefore, proves that the components of the whole extract work in synergy to evoke an overall positive response in the plant system where each component might act on various metabolic networks either independently or interactively [107,120] (Figure 1 and Figure 3).

Plants treated with seaweed extracts have shown generally improved nutrient acquisition capabilities and improved growth and vigor. *A. nodosum* extract-treated rapeseed plants showed an increase in nitrogen and sulfur acquisition. Transcription studies showed that this was due to an overexpression of the BnNRT1.1/BnNRT2.1 and BnSultr4.1/BnSultr4.2 genes which encode root transporters associated with the uptake of nitrogen, iron and sulfur [121]. This was explained as a result of the upregulation of genes coding sulphate, iron and nitrate transporters. Plant nutrient acquisition increased in seaweed extract treated plants was also evidenced by an increase in the transcription or activity of nutrient transporters in the plant membrane [122]. A study conducted in spinach using *A. nodosum* showed increased biomass, protein content, chlorophyll and carotenoid content, flavonoids and phenolics, and increased antioxidant activity. The increase in biomass was correlated with an increase in the expression of the GS1 gene involved in nitrogen integration. The increase in chlorophyll content was related to an increase in the expression of betaine aldehyde dehydrogenase and choline monooxygenase. The increase in chitinase activity, phenolics, and flavonoids was attributed to an increase in the expression of glutathione reductase, thylakoid bound ascorbate peroxidase APX, and monodehydroascorbate reductase. These genes were linked to the phenylpropanoid and flavonoid pathways which are known to stimulate growth and enhance overall nutrition [123]. A microarray study conducted on rapeseed showed the differential expression of 724 and 612 genes after 3 and 30 days of treatment, respectively. Ontologies of these genes showed involvement in carbon, sulfur, and nitrogen metabolism, cell metabolism, photosynthesis, and various metabolic pathways involved in phytohormone synthesis, fatty acids, plant development, and the transport of ions. Correlation analyses showed a link between differentially expressed genes and increased S, N, and sulfate as well as enhanced chlorophyll content and numbers of chloroplasts and starch granules, all of which led to improved growth. This led to the further hypothesis of an enhancement in carbon assimilation and the synthesis of starch which are linked to the enhanced expression of Rubisco and carbonic anhydrase [124]. A study conducted on tomato and bell pepper also demonstrated the effects of different seaweed extracts on major hormonal biosynthesis genes. Studies further revealed that seaweed extracts were able to significantly upregulate genes involved in auxin (*IAA*), gibberellin (*Ga_2_O_x_*), and cytokinin (*IPT*) biosynthesis which could be correlated to the increased plant growth effects [5,13].

Seaweed extracts also aid plants to withstand harsh environmental conditions such as cold, drought, and salinity. Application of *A. nodosum* extracts protected *Arabidopsis* plants from induced cold stress by augmented chlorophyll content, possibly due to downregulation of chlorophyll degradation genes (AtCLH1 and AtCLH2). In addition, upregulation was observed with the transcription factor DREB1A and the COR78/RD29A genes encoding cryoprotection of chloroplast stromal protein, which are key regulators for cold stress tolerance [125]. The increase in proline, soluble sugar, and unsaturated fatty acid content was also related to increased tolerability to cold stress. Proline accumulation was supported by upregulation of 5CS1 and P5CS2 which are responsible for proline biosynthesis and the downregulation of ProDH, a gene involved with proline degradation. The increase in soluble sugars was linked to the upregulation of polysaccharide degradation genes 9SEX1 and SEX4, upregulation of carbohydrate biosynthesis genes (GOLS2 and GOLS3), and the downregulation of sucrose degradation genes. In addition, the same study also reported the upregulation of the gene DGD1 which is involved in the synthesis of galactolipid that is known to play a role in cold stress tolerance. A study using tobacco cultures treated with *A. nodosum* extracts revealed the same trend wherein the treated cultures showed significant levels of tolerance to freezing stress. This trait was attributed to the upregulation of key freezing tolerance genes such as galactinol synthase 2, pyrroline 5-carboxylate synthase, and acetyl-CoA carboxylase [126].

Interestingly, seaweed extracts have been able to mitigate significantly the drought stress in plants, though its mechanism of action is still hazy at the most. Studies showed that seaweed extract treated plants under drought conditions have maintained higher relative water content, improved water use efficiency, stomatal conductance, and transpiration rate reduction. The increased expression of abscisic acid-responsive genes (At5g66400 and At5g52310) was noted in seaweed extract treated plants. The findings also revealed the optimal conservation of photosystem II (PSII) photochemistry and a boosted nonphotochemical quenching. Increased expression of the antioxidant coding genes At5g42800 and At1g8830 was also reported, which led to oxidative damage prevention to photosystem II in treated plants [127]. Reduced wilting and an increase in recovery rate, greater stomatal conductance, and augmented reactive oxygen scavenging activity were observed in soybean plants under drought stress when treated with seaweed extracts. Genes involved in ABA catabolism (GmCYP707A1a and GmCYP707A3b) were implicated in these results. The ABA-inducible GmDREB1B as well as the BURP domain protein-encoding GmRD22 were also significantly upregulated in seaweed extract treated plants. Interestingly, genes involved in the detoxification of ROS were also significantly upregulated (GmGST, GmBIP, and GmTP55) [70]. Similar drought stress mediation was also recorded in tomato in response to seaweed extract treatment. The lipid peroxidation levels were much lower compared to untreated plants under water stress. Furthermore, quantities of glucose, proline, and sucrose were significantly greater in treated tomato plants. Up to an 8-fold increase in the *tas14* gene was seen in treated plants which encode for dehydrin proteins responsible for abiotic stress alleviation [128]. 

Apart from cold and drought stress, salinity stress also poses great threats to the agriculture sector and luckily, seaweed extracts can also alleviate this problem. A microarray analysis done on *Arabidopsis* plants presented the upregulation of 257 genes under salt-induced stress when treated with extracts. Highly upregulated genes included the late embryogenesis abundant 3 family and transcription factor Circadian Clock Associated 1 which have been linked to abiotic stress tolerance [120]. Other key genes induced were glutathione S-transferase-encoding, dehydration-responsive protein transcripts, ABA signaling genes (At5g62490 and At4g15910), and late embryogenesis abundant 1 and 2, which are all key players in the fight against abiotic stress [129]. The role of micro RNAs (miRNAs) has also been shown to impart drought stress alleviation as well as other abiotic stresses [70]. Over 106 miRNAs were significantly expressed in *Arabidopsis* under salinity stress when treated with seaweed extracts, some of which aid in both salinity as well as drought tolerance. Treated plants also showed lower sodium and higher phosphorous levels under NaCl-induced stress conditions. Additionally, the genes involved in phosphate starvation regulation (ath-miR399a, ath-miR399b, ath-miR399c-3p, and ath-miR399c-5p) were significantly reduced with seaweed extract treatment [130]. An in vitro study conducted in tomato and sweet pepper showed that under NaCl-induced stress, seedlings were able to overcome excessive oxidative damage when pre-treated with seaweed extract. This was correlated with the significant increase in the activity of antioxidant enzymes including ascorbate peroxidase and catalase [62]. A similar study conducted in biscuit grass under saline conditions showed the enhancement of lipid peroxidation and an increase in the activities of catalase, superoxide dismutase, and ascorbate peroxidase which ultimately led to a reduction of hydrogen peroxide levels in treated plants [131].

Apart from abiotic stresses, seaweed extracts have been able to enlist a number of defense mechanisms in plants against biotic stressors. The major cell wall polysaccharides of seaweeds such as ulvans, laminarins, and carrageenans and their derived oligosaccharides have been shown to confer some of these resistant responses in plants [55]. These bioactive molecules are known to induce an oxidative burst and various defense pathways mediated through salicylic acid, jasmonic acid, and ethylene. This cascade of reactions then leads to the accumulation of pathogenesis-related proteins (PR proteins), various defense enzymes such as chitinases and glucanases, and an increase in phenolic compounds which led to greater protection against a broad spectrum of pathogens. An extract from *Ulva* spp. applied to barrel clover under *Colletotrichum trifolii* infection led to an increase in the activity of defense enzymes such as phenylalanine ammonia-lyase, chitinase, chalcone synthase, and isoflavone reductase. This increase in defense enzymes led to the downregulation of genes responsible for carbon and nitrogen metabolism. This systematic response is quite normal as the plants use more resources to deploy the required pathways to protect itself under pathogenic attack. Interestingly, in vitro assays showed no direct inhibition of *C. trifolli* by seaweed extract application, suggesting that the defense mechanism is solely plant-based, through elicitation of key defense systems [132]. Similar studies conducted using *Ascophyllum nodosum*, *Sargassum vulgare*, and *Acanthophora spicifera* extracts also proved the absence of antimicrobial activity at the recommended concentrations [4,31]. A study conducted in tomato and sweet pepper recorded reduced infection levels by *Xanthomonas campestris* pv. *vesicatoria* and *Alternaria solani,* both in greenhouse and field conditions, following spray treatment by *A. nodosum*, *Sargassum vulgare*, and *Acanthophora spicifera* extracts. The extracts were able to significantly upregulate genes involved in SA and/or JA and ET mediated defense signaling including *PR1-a*, *PinII,* and *ETR-1*, respectively, as well as an increase in defense enzyme activity (phenylalanine ammonia-lyase, peroxidase, polyphenol oxidase, chitinase, and β-1,3-glucanase) [5,13]. Furthermore, the above studies also demonstrated no direct antimicrobial effects of the seaweed extracts, echoing the findings of Cluzet et al. [132]. The studies by Ramkissoon et al. [25] and Ali et al. [31] revealed an interesting observation about the elicitation of defense pathways by the types of seaweed extracts. In these studies, it was demonstrated that the treatment by extracts from the brown seaweeds (ex. *Sargassum* and *Ascophyllum*) had no significant effect on the SA pathway which was evidenced by no significant upregulation of *PR-1*a transcripts in treated plants. However, brown seaweed extracts caused significant upregulation of both *PinII* and *Etr*-1 based defense pathways (JA and Etr pathways) which are primarily ISR type of responses. On the other hand, the red seaweed (*Acanthophora spicifera*) extract, induced the expression of the *PR-1*a gene at early stages (12–48 h after treatment), and when the level declined (at 48 h), it led to the upregulation of the *PinII* and *Etr*-1 gene transcripts at later time points (72–96 h). This kind of harmonized modulation of pathways is a very interesting observation which was reported for the first time by these studies. These observations also demonstrated the obvious antagonistic activities of SA, JA, and ET pathways [133]. The brown seaweeds comprise of 17–45% alginates [134] and 5–20% fucoidan [135] and laminarins, which have been linked to high bioactivity when applied to plants [9]. These major carbohydrate components of the brown seaweeds are known to negatively affect SA signaling [136,137]. This could be the reason for the low accumulation of the *PR-1*a gene transcripts in brown seaweed extract treated plants. The studies by Ramkissoon et al. and Ali et al. [25,31] have confirmed that the main trigger of defense by brown seaweed extracts in plants was via the JA and ET mediated defense signaling pathways. However, the red seaweeds contain a variety of carrageenans [lambda (λ), kappa (κ) and iota (ι), mu (μ), nu (υ) and theta (θ)], and all of them can act as elicitors [138] for SAR type responses. These carrageenans, which are not as sulfated, mainly elicit a SA defense-mediated signaling or systemic acquired resistance (SAR). Whereas the presence of higher levels of sulfated carrageenans can activate only induced systemic resistance (ISR)-type responses. These active polysaccharides present in seaweed extracts can elicit unique responses including upregulation of various PR proteins, defense genes and enzymes belonging to different pathways leading to the development of induced resistance and preconditioning of the plants. Therefore, compositional differences of seaweed extracts related to the species could be the plausible reason for the differential responses on defense pathway induction, as supported by previous studies [25,31]. 

The presence of anti-juvenile hormonal compounds in the seaweed (*Padina pavonica*) extract which significantly disrupted the cotton stainer bug (*Dysdercus cingulatus*) development stages at the germ bud or even at the stage of blastokineis [139]. Additionally, the phenolic compounds present in seaweed extracts were able to significantly reduce root knot nematode infestation in banana [140]. However, this is just a brief snapshot of the entire eliciting process of seaweed extracts and further studies are yet to be conducted to reveal the complexity of mechanisms.

Altogether, these results show the major properties associated with seaweed extracts, in that they cause elicitation of a cascade of metabolic networks to awaken in plants, thereby giving optimal protection against both biotic and abiotic stresses. Furthermore, it should also be stressed that these extracts perform better as a whole product rather than as fractions since the components interact together to produce synergies that act comprehensively on the plant system. Therefore, the observed effects are not due to a single defense pathway or a specific set of genes but due to the interaction of multiple genes in an organized, and harmonic fashion.

## 7. Effect of Seaweed Extracts on the Plant and Soil Microbiome Dynamics

Plant surfaces including roots and leaves constitute the rhizosphere and phyllosphere where interactions between plant and microbes are highly active and vibrant. These microbial-based interactions have a profound influence on plant growth. Apart from external surfaces, the plant internal surfaces are also colonized by microorganisms wherein active interactions also significantly affect plant growth and productivity. Plant roots, leaves, and internal surfaces exude or release several water-soluble compounds such as amino acids, sugars, and organic acids, and these supports or influence the growth of a variety of microorganisms [143]. 

Application of biostimulants, fungicides, and other inputs over the foliage or soil has a significant influence on the exudate composition of the plant’s external and internal surfaces. High levels of exudates in the rhizosphere activate a plethora of microorganisms. The composition and pattern of root exudates affect the population structure, size, and activity. As expected, the application of seaweed extracts to soil and foliage has significant effects on the rhizosphere and phyllosphere microbiome patterns. The healthy and productive growth of plants treated with seaweed extracts has also been suggested to be influenced by the microbiome effect conditioned by the extracts [10]. Therefore, seaweed extracts could potentially enhance PGP (plant growth promotion) traits of rhizospheric microbes.

A study was conducted on tomato and pepper plants in the greenhouse using *Ascophyllum nodosum* extract (ANE) to test its effect on bacterial and fungal communities in rhizospheric soils. Amplicon sequencing targeting fungal ITS and bacterial 16S rRNA genes to determine microbial community structure changes was conducted. The results showed a significant increase in plant growth parameters including root, shoot, and fruit biomass by ANE. Interestingly, the species composition of both fungi and bacteria on the roots and soil had significant variations (*b*-diversity) between the ANE treatment and the control. These variations were mostly inclined towards several beneficial groups of microbes growing in the rhizosphere, which could have had a significant influence on the growth of plants [144]. Similarly, when maize plants were applied with *Ascophyllum nodosum*-fermented seaweed fertilizer, there was a significant effect on the rhizosphere microbiome. The relative abundance of the dominant phyla varied, and the bacterial α-diversity was significantly influenced by the seaweed fertilizer application. Additionally, the enzymatic activities of dehydrogenase, nitrite reductase, urease, and cellulase in the soil were significantly increased up to 13 days after the application of ANE to the maize rhizosphere soil. A seaweed extract biostimulant, based on *Lessonia nigrescens* and *Lessonia flavicans* applied to replant soil of *Malus hupehensis* seedlings, significantly increased the soil activity of invertase, urease, proteinase, and phosphatase enzymes in comparison with the control. T-RFLP analysis results showed that the soil fungal community had been significantly altered after the application of the seaweed extract [145]. The biolog analysis of the microbial metabolic activity of strawberry plants’ rhizosphere soil, as well as functional diversity, colony counts, and soil respiration showed significant increases in response to *Ascophyllum nodosum* extract treatment under greenhouse and field conditions [132]. Although those effects are of major importance, the underlying microbiome effects and plant-microbiome interactions are largely under investigated. There is a growing need to use metagenomics tools to fully reveal the degree of microbial community discrepancy induced by seaweed extract application. These results will deepen our understanding of plant-microbe interactions in seaweed extract treatments and would help rationalize their use in sustainable agricultural production. 

## 8. Bioactive and Elicitor Components of Seaweed Extracts

Seaweed extracts comprise of a wide array of bioactive substances that elicit and directly promote plant growth and defense reactions [3]. Some of these substances involved in various metabolic pathways include polysaccharides, plant growth-promoting hormones, fatty acids, sterols, carotenoids, oxylipins, minerals, peptides, amino acids and proteins, lipids, polyphenolics phlorotannins, all of which are biologically active [3,16]. These substances in the extracts vary differently based on the class and species of seaweed as well as the type of extraction method utilized.

Seaweeds contain many different polysaccharides, whose type, quantity, and chemical structure depends on the species of the seaweed and ecological conditions. Seaweeds normally contain polysaccharides up to 76% of dry weight but the content also shows seasonal variations. Among many different algal polysaccharides, the most important types are galactans, fucoidan, laminarin, and alginates, and most of these are proportionally represented in the seaweed extracts. The methods of extraction do have a great influence on the composition of the seaweed extracts. Normally during the extraction process, the complex molecules including polysaccharides are converted into oligomers which are highly bioactive in plants. In the same way, the small molecules like hormones may be significantly degraded [11]. Seaweed extracts contain different kinds of carotenoids, which are very strong antioxidants. The extracts also contain phenolic compounds including phenolic acids, flavonoids, isoflavones, cinnamic acid, benzoic acid, quercetin, and lignans. Algal extracts are known to contain various minerals since seaweeds basically bioaccumulate minerals found in the seawater. Seaweed extracts also have phytohormonic substances including cytokinins, gibberellins, auxins, abscisic acid, and betaines [142]. The effect of these substances on crops depends on the type of plant, its receptor mechanism, and the type of application used, specifically whether foliar, root feeding, or a combination of both [12]. Figure 2 and Table 3 highlight the important compounds present in seaweed extracts which all work harmoniously to promote plant growth and induce defense mechanisms. 

**Table 3:** Major bioactive components of seaweed extracts.

Bioactive Compounds	Chlorophyceae (Green)	Rhodophyceae (Red)	Phaeophyceae (Brown)	Reference
Polysaccharides	- Amylose, amylopectin - Cellulose - Glucomannans - Inulin - Laminaran - Ulvans - Sulfated mucilages - Xylans - Pectin - Mannans	- Agars, agaroids - Cellulose - Mannans - Carrageenans - Complex mucilages - Furcellaran - Glycogen (floridean starch) - Xylans - Rhodymanan	- Alginates - Cellulose - Heteroglucans - Fucose - Fucoidans - Glucuronoxylofucans - Laminarans - Lichenan-like glucan	[55,137]
Plant Growth Regulators	- Cytokinins - Auxins - Gibberellins - Abscisic acid (ABA) - Indole-3-acetic acid (IAA) - Ethylene - Brassinosteroids - Jasmonates - Salicylic Acid - Strigolactones - Zeatin - Kinetin - 6-benzyl amino purine (BAP)	- Cytokinins - Auxins - Gibberellins - Abscisic acid (ABA) - Indole-3-acetic acid (IAA) - Ethylene - Brassinosteroids - Jasmonates - Salicylic Acid - Strigolactones - Zeatin - Kinetin - 6-benzyl amino purine (BAP)	- Cytokinins - Auxins - Gibberellins - Abscisic acid (ABA) - Indole-3-acetic acid (IAA) - Ethylene - Brassinosteroids - Jasmonates - Salicylic Acid - Strigolactones - Zeatin - Kinetin - 6-benzyl amino purine (BAP)	[16,146,147]
Betaines	- Glycine - γ-Aminobutyric acid - δ-Aminovaleric acid - Laminine	- Glycine - γ-Aminobutyric acid - δ-Aminovaleric acid - Laminine	- Glycine - γ-Aminobutyric acid - δ-Aminovaleric acid - Laminine	[148,149]
Sterols	- Ergosterol - Isofucosterol - Clerosterol - Clionasterol	- Cholesterol - Cholesterol derivatives - Fucosterol - β-sitosterol - Campesterol	- Fucosterol - Fucosterol derivatives - Cholesterol - Campesterol - Stigmasterol	[150,151,152,153]
Carotenoid	- β-carotene - Lutein - Violaxanthin - Antheraxanthin - Zeaxanthin - Neoxanthin	- β-carotene - α -carotene - Zeaxanthin - Lutein	- Fucoxanthin - β-carotene - Violaxanthin	[154,155]
Minerals	- Macro (C, Cl, Fe, Mg, P, K, Na and S) - Micro (B, Cr, Co, Cu, F, Gr, I, Mn, Mo, Ni, Se, Si, S, Tn, W, V, Zn)	- Macro (C, Cl, Fe, Mg, P, K, Na and S) - Micro (B, Cr, Co, Cu, F, Gr, I, Mn, Mo, Ni, Se, Si, S, Tn, W, V, Zn)	- Macro (C, Cl, Fe, Mg, P, K, Na and S) - Micro (B, Cr, Co, Cu, F, Gr, I, Mn, Mo, Ni, Se, Si, S, Tn, W, V, Zn)	[156,157,158]
Polyphenolics and Phlorotannins	- Bromophenols - Flavonoids	- Bromophenols - Flavonoids - Phenolic terpenoids - Mycosporine-like amino - Acid	- Bromophenols - Flavonoids - Phenolic terpenoids - Phloroglucinol - Eckol - Dieckol	[159,160]
Lipids	- Glycolipids - Betaine lipids - Non-polar glycerolipids (neutral lipids) - Mannose and rhamnose containing glycolipids	- Sulfur-containing phospholipids - Phosphatidyl sulfocholine - Glycolipids - Betaine lipids - Non-polar glycerolipids (neutral lipids) - Sulfonoglycolipid crassiculisine	- Glycolipids - Betaine lipids - Non-polar glycerolipids (neutral lipids) - Unusual lipid class	[153,161,162]
Oxylipins	- Hydroxy and hydroperoxy fatty acids - (FAs) Coalital (C10-oxylipin) - Epoxy alcohol - Hydroxy and hydroperoxy FAs - 13-oxo-trideca-9,11-dienoic acid, (2Z) - pentane, pentanol, hexanal	- Hydroperoxy FAs - Hydroxy FAs - Diols - Epoxy FAs - Prostaglandins - Leukotrienes - Cyclopropyl hydroxyeicosanoids - Eicosanoids - Hepoxilin like metabolite - Polyneuric acid	- Ecklonialactones - Egregialactones - Carbocyclic eiseniachlorides, eiseniaiodides - and bicyclic cymathere ethers - Hydroxy-, hydroperoxy FAs	[151,160,161,162,163]
Protein, peptides, and amino acids	- Histidine, Isoleucine, Leucine, Lysine, methionine, Phenylalanine, Threonine, Tryptophan, Valine, Cysteine, Arginine, Aspartic acid, Glutamic acid, Alanine, Glycine, Proline, Serine, Tyrosine and Alanine - Taurine - Domoic acid - α-Kainic acid	- Histidine, Isoleucine, Leucine, Lysine, methionine, Phenylalanine, Threonine, Tryptophan, Valine, Cysteine, Arginine, Aspartic acid, Glutamic acid, Alanine, Glycine, Proline, Serine, Tyrosine and Alanine - Taurine - Domoic acid - α-Kainic acid	- Histidine, Isoleucine, Leucine, Lysine, methionine, Phenylalanine, Threonine, Tryptophan, Valine, Cysteine, Arginine, Aspartic acid, Glutamic acid, Alanine, Glycine, Proline, Serine, Tyrosine and Alanine - Taurine - α-Kainic acid	[164,165,166]

## 9. Seaweed Extract as an Input for Integrated Crop Management Program: A Paradigm Shift towards Sustainable Agriculture

Due to the intensification of agriculture globally, there is a heavy reliance on the use of synthetic chemical inputs in agriculture. Most of these synthetic chemical inputs used however have their drawbacks, especially when overused. Although optimum usage leads to overall higher returns, it can cause toxic and long-lasting unfavorable effects on the environment and humans, especially by improper and over-usage. These drawbacks may cause an increase in pesticide resistance, run-off into the environment causing serious problems such as eutrophication, water contamination, leftover residue causing harm to humans and animals, and overall increases in production costs [167]. Apart from those effects, there is the added danger of non-target effects. Studies have shown that constant overuse of pesticides and chemical fertilizers has led to the downfall of non-target beneficial organisms which ultimately affects the entire food chain, thereby affecting overall diversity [168]. The World Health Organization (WHO) reports approximately 25 million cases of acute occupational pesticide poising in developing countries [169]. Many of these pesticides are now being tightly regulated, especially in developed countries since studies have shown their high persistence in the environment and highly toxic effects on humans such as carcinogenicity, hormonal imbalances, spermatotoxicity, teratogenicity [170,171].

Many countries have come forward with initiatives to minimize the persistent ill effects of chemicals in agriculture by imposing laws and implementing intensive management programs. These include basically integrated management methods wherein application of synthetic chemicals is kept at a minimum, and more emphasis is given to enhance natural systems of protection using natural enemies and non-chemical, biological, and organic inputs [172]. This is the era wherein implementation of holistic approaches or systems including integrated crop management (ICM), integrated nutrient management (INM), integrated disease management (IDM), an integrated pest management (IPM) are under imperative practice [173]. 

In light of the integrated management methods, scientists and farmers alike are looking for ecologically safe alternatives, one of which in the forefront is seaweed extract biostimulants/products [172]. The previous chapters highlighted the positive impacts of seaweed extracts on the overall net return of crops. The integrated management approach in agriculture aims at applying more than one method in order to control disease outbreaks in crops, and by that, the use of seaweed extracts could be a preferred component due to its multiple modes of action. Their non-toxic nature and multiple beneficial roles to the crop and environment are the major highlights for being considered as green inputs. Feasibility studies have shown that farmers are willing to use seaweed extracts and products as “green alternatives” within their existing cropping scheme [13]. New studies have shown that farmers who used less than half their usual chemical inputs per crop in conjunction with seaweed extract, received higher returns from the produce. Studies done in the tropics showed that sweet pepper and tomato plants treated with extracts of either *A. nodosum*, *S. vulgare*, or *A. spicifera* in rotation with safe fungicides had the lowest disease levels and the overall highest total marketable yield compared to treatments with seaweed extract alone or fungicide alone. It has been speculated that this positive effect of seaweed extracts could have been a result of their multiple beneficial actions on the crop host ultimately leading to lower disease and pest levels and greater yields [4,28,31].

A five year-long research study by UWI-St. Augustine entitled, “Developing sustainable disease management strategies to improve vegetable production towards self-sufficiency and food security in the Caribbean region” utilized many aspects of IDM components in which seaweed extracts were used as a prominent input. The results showed that integrating various inputs in the cultivation of tomato, pumpkin, pepper, and cowpea lead to significant reductions of major diseases as well as significant improvements in the total marketable yield and while reducing the incidence of pests and diseases [174]. These studies, therefore, demonstrated and certified the essential role of seaweed extract spray as an organic component for widespread use in crops. By increasing the rate of use of seaweed extracts and other organic inputs, the standard rate of application of chemicals was reduced by two-thirds without negatively impacting yields [10]. Most of the seaweed-based products are classified as biostimulants, organic nutrients, or plant boosters and were considered as organic inputs. This contributes to the extensive use of seaweed products to crops certified for organic production as well as environmental-friendly production or for regular use under integrated crop management systems [12,174]. 

## 10. Challenges and Opportunities for Seaweed Biomass Valorization and Development of Novel Agricultural Inputs

The utilization of seaweed biomass as a resource for deriving novel products for use in agricultural industries, while appears challenging, has however created a fair number of opportunities for scientific knowledge generation and innovation. Though seaweeds are a highly renewable biomass, generally, there are significant challenges in harvesting, processing, and storage of seaweed biomass. Additionally, seasonal growth and inflow of seaweed biomass force industries to focus on efficient harvesting techniques, quick processing, and storage methods. Sustainable harvesting methods have been developed and proven successful for brown kelp, and, therefore, there is a great need for optimizing the harvesting of other important seaweeds. For the well-known kelp species, efficient harvesting of biomass during growth seasons has helped preserve the native growth and ecological biodiversity. Further to this, wet and dry processing and preservation methods were optimized for efficient processing, storage, extraction, and biotransformation [175].

There are various extraction protocols developed which employ physical and chemical methods to efficiently extract the biomolecules with minimum loss to their conformation and bioactivity [122]. The active molecules can either be purified or used as whole extracts for agricultural purposes. Many biostimulant based formulations have been developed using seaweed extract as a component or as the complete ingredient [14]. But still, extraction protocols need to be optimized for some of the promising seaweeds. With regard to formulations, there are only liquid, dispersible, and soluble solids available. Not many successful efforts have been reported for developing novel commercial formulations for use in traditional and protected crop production systems. Further, developing composite formulations or value-added formulations incorporating a mixture of seaweeds, other biological components including live microorganisms, plant extracts, and biopesticides is still a technological gap that needs to be bridged [12]. 

While there are several potential beneficial effects of seaweed products, contrastingly there are also some undesirable side effects posed by the seaweed-based products which need to be carefully dealt upfront. For instance, the contamination of seaweed biomass by heavy metals and persistent organic pollutants threaten their widespread use in agricultural applications [176]. Many of the seaweeds tend to absorb pollutants normally found in the substrate water. Excessive contamination by pollutants can challenge their use for processing and extensive applications in agriculture. Therefore, technologies are being developed for clarification of pollutants prior to or after processing [177,178].

The imposition of strict quality requirements on seaweed biomass is not practically feasible for agricultural products including extracts and composts. This is due to the inherent variations in the species, their growth habits, and habitats, geographical location, environmental and climatic conditions. It is, therefore, quite hard to maintain consistent quality as pure seaweed extract is fully biological. Quality could only be optimized by blending batches of extracts and by necessarily adding adjuvants without compromising their biological status and label. Often, the seaweed products, particularly liquid formulations become less stable over time. This difficulty needs to be addressed by finding suitable stabilizers and additives to enhance the shelf life and stability of quality. 

Seaweed products used in agriculture are generally proven environmentally safe and considered to be organic components, if the formulation contains a maximum level of natural ingredients. However, there are also several spurious products circulating in the market in the name of “seaweed organic biostimulants” which contain only a small fraction of seaweed-derived components but comprise mainly of synthetic mineral nutrients and chemicals. These were being sold as biofertilizers or biostimulants which creates confusion among users. This mandates imposition of strict quality standards to correctly identify, certify, and label products with the originality of their organic composition. 

Though the seaweed extracts evoke multiple responses in plants to counter biotic and abiotic stresses, much of the genetic, physiological, and biochemical mechanisms are yet to be unraveled, which requires comprehensive and systematic research investigations. Further, most of the research tends to be focused on greenhouse and controlled environment trials. Rigorous experimentation at the field level and data collection at multi-crop, multi-seasonal, multi-locational and geographical situations are needed before recommendation to growers. The delivery of seaweed products is currently predominantly limited to foliar and soil application. Soil application is often expensive as it requires high levels and multiple applications, which is a limitation. Methods of the application still need to be worked out which favors optimal applications without burdening the overall cost of production. There are also some challenges in labeling the seaweed extract-based formulations due to their multiple activities and functions. The use of labels “biostimulant/biofertilizer” for seaweed extracts are becoming obsolete or out of context given their multiple activities. This creates practical difficulty in the classification of products in the commercial sense. In terms of usage, seaweed extract should be considered as an input suitable for integration and complementation with other inputs used in agriculture. This also requires extensive field-based integrated crop production evaluation trials in each crop in different seasons and locations. Ideally, the use of seaweed extracts should minimize the application of chemical inputs and thereby vertically reducing the chemical load and cost [13]. 

The seaweed biomass can provide a rich resource for multiple applications and each one of them has matured into industries with a high level of contribution to the micro and macroeconomy. The extracts apart from their phytostimulant value, they act as a resource for the extraction of value-added biomolecules including polysaccharides and several industrial, pharmaceutical, nutraceutical, feed, and food supplements and products. The final residue after extraction can be used as a soil amendment, animal feed supplement, compost base, planting mix, biochar base, or even construction materials (ex. particleboard, concrete, etc.) [179]. Therefore, seaweed biomass is an excellent resource for developing multiple products that are both environmentally neutral and sustainable thereby promoting recyclability and a circular methodology and circular economy.

Since seaweed biomass accumulation and bycatch happens at shallow marine habitats of countries with a sea line, anglers and the beachline communities are the mainly affected parties. The recent surge in *Sargassum* accumulation in the Caribbean region, particularly in the island states, has resulted in many of these countries incurring significant losses to their tourist economy [180]. However, the development of successful and sustainable methods for the valorization of the biomass of *Sargassum* and other seaweeds could generate diverse opportunities for multiple economic activities. The methods so far developed can offer some solution to this problem. In this way, the serious ecological problem of seaweed biomass accumulation can be collectively managed by proper resource utilization and transformation of biomass [10]. The fishermen and the beach communities can be employed or involved in biomass collection and bioprocessing in a systematic manner. These ideas hold promise for developing small cooperative or community-based industries as well as larger-scale operations involved in processing and valorization of seaweeds for deriving multiple products through circular economy.

## 11. Conclusions

Generally, the positive impacts of seaweed extract-based biostimulants on crop production and the environment warrant their prescription for applications in different cropping systems. Published reports so far confirmed the positive effects on plant growth, vigor, enhanced tolerance to pests, diseases, and abiotic stresses, as well as an overall improvement in plant productivity. The summary of the effects of seaweed extracts on crops is depicted in Figure 3. Published reports also highlighted the improvement in the nutritional quality of seaweed extract treated crops including increased antioxidant contents, which enhances the attractiveness of seaweed products for use in crop production. The positive effects of the seaweed products are dependent on the type of the seaweed resource, quality, and composition of the extract, and the method, concentration, and frequency of application. All the enhanced growth effects have been observed only with the whole extract, which underlines the very interactive nature and synergistic activity of the seaweed extract components on plant growth and functions. However, the synergistic activities and interactions of biomolecules and their molecular functions on the plants are largely unresolved due to its complexity. There is strong evidence to support the role of seaweed extract applications in altering the microbiome of the rhizosphere and phyllosphere. Further studies into this area should verify the effect of introduced and native beneficial microbes in order to improve the microbial plant growth promotion. There is also a necessity to understand the basic level of communication between the plant and microbial systems’ interface as influenced by seaweed extracts. Emerging studies also support the compatibility of seaweed extracts and products with other agricultural chemical and non-chemical inputs. Although using seaweed extract as a sole input or standalone method may not be sustainable, it is ideal to use a minimum dose of pesticides which can synergize the seaweed effects and benefit the overall crop production phenomenon. The seaweed extract formulations are available as liquid concentrates which limits their shelf life. Efforts are needed to produce user-friendly and stable solid formulations that can have improved shelf-life. The soil application of seaweed extract remains economically challenging due to the high levels of input needed. Alternative means including root feeding, foliar misting, and drip application need to be optimized for the crops and growing systems. Although seaweed biomass is renewable, due care should be taken to prevent over-exploitation and disturbance to the marine or coastal biodiversity. The recent excessive inflow of Sargassum weed in the pacific and Atlantic currents are posing serious challenges to the marine and coastal biodiversity. Methods for valorisation and transformation of this seaweed into value added and novel products are still at the stages of infancy. On the other count, commercial culturing of economically important seaweeds for development of novel biostimulants or commercial products would be essential in order to conserve the seaweed resources and prevent overuse and extinction from their natural ecosystems. Considering the current focus and necessity for organic and environmentally sensible agriculture, the need for more effective organic inputs is ever growing. Being renewable bioresources, seaweeds and their bioproducts can certainly provide multiple organic inputs to the levels to meet the ever-growing crop needs. Research and development, therefore, should continue in these directions to maximize the potential utility of seaweeds and their products in sustainable agriculture. 

## Figures and Tables

**Table 1:** List of important seaweed species with documented biostimulatory activities.

Phaeophyceae	Rhodophyta	Chlorophyta
***Ascophyllum nodosum***	*Macrocycstis pyrifera*	*Ulva lactuca*
***Ecklonia maxima***	*Porphyra perforate*	*Enteromorpha prolifera*
***Durvillea antarctica***	*Nereocystis* spp.	*Caulerpa paspaloides*
***Durvillea protatorum***	*Cyanidium caldarium*	*Ulva armoricana*
***Fucus vesiculosus***	*Gelidium serrulatum*	*Codium Liyengarii*
***Sargassum* spp.**	*Acanthophora spicifera*	*Codium tomentosum*
***Hydroclathrus* spp.**	*Kappaphycus alvarezii*	*Caulerpa sertularioides*
***Ralfsia* spp.**	*Gracilaria edulis*	
***Laminaria digitata***	*Gracilaria dura*	
***Cystoseira myriophylloides***	*Laurencia johnstonii*	
***Fucus spiralis***		
***Padina pavonica***		
***Fucus gardneri***		
***Durvillaea antarctica***		
